# High Prevalence of Myositis-Specific and Associated Antibodies in Patients with Pulmonary Hypertension

**DOI:** 10.3390/diagnostics14141471

**Published:** 2024-07-09

**Authors:** Rachid Tobal, Judith Potjewijd, Daan van Doorn, Vanessa van Empel, Jan Damoiseaux, Pieter van Paassen

**Affiliations:** 1 Department of Internal Medicine, Division of Nephrology and Clinical and Experimental Immunology, Maastricht University Medical Center, 6229 HX Maastricht, The Netherlands; daan.van.doorn@mumc.nl (D.v.D.); p.vanpaassen@maastrichtuniversity.nl (P.v.P.); 2 Department of Cardiology, Maastricht University Medical Center, 6229 HX Maastricht, The Netherlands; vanessa.van.empel@mumc.nl; 3 Central Diagnostic Laboratory, Maastricht University Medical Center, 6229 HX Maastricht, The Netherlands; jan.damoiseaux@mumc.nl

**Keywords:** pulmonary hypertension, myositis-specific antibodies, myositis-associated antibodies, immunology, auto-antibodies

## Abstract

Pulmonary hypertension (PH) is a serious condition linked to immune-system dysfunction. Myositis-specific/associated antibodies (MSAs/MAAs) play a role in idiopathic inflammatory myopathy (IIM) and interstitial lung disease (ILD), but their significance in PH remains unclear. We believe the presence of these antibodies may be underestimated. This study analyzed adult PH patients without pre-existing IIM for MSA/MAA prevalence using a line-blot assay. We compared PH patients with and without ILD signs to a cohort clinically suspected of IIM/ILD (*n* = 558). Our PH cohort (*n* = 121) showed a significantly higher prevalence of overall weak positive MSAs/MAAs and positive overlap syndrome-associated MAAs than the suspected IIM/ILD group (*p* < 0.001). Notably, MSAs/MAAs were found in PH patients both with and without ILD, though more prevalent in those with ILD. Anti-synthetase and anti-overlap syndrome antibodies were the most common. Our study is the first to systematically show a high MSA/MAA prevalence in PH without IIM presentation. This highlights the need to consider PH when diagnosing MSA/MAA-associated conditions. We recommend MSA/MAA screening for newly diagnosed PH, especially in those with ILD, for early detection and potential immunomodulatory treatment. Further research should explore the link between MSAs/MAAs and PH, and the value of monitoring patients with weak MSA/MAA positivity over time.

## 1. Introduction

Pulmonary hypertension (PH), a severe condition with high morbidity and mortality, significantly impacts patients’ quality of life [1]. Elevated pressure within the pulmonary vasculature leads to right-sided heart failure and potential death. Vascular remodeling within the pulmonary arterioles is a hallmark of pre-capillary PH, while post-capillary PH arises from changes in the venous vasculature, often diagnosed in patients with left-sided heart disease. Right heart catheterization (RHC) distinguishes these subtypes. PH is a hemodynamic diagnosis and has diverse etiologies. This is illustrated by the fact that the World Health Organization (WHO) has classified PH into five different clinical subtypes, indicating similarities in pathobiology, although overlap between classes remains [2]. Immune dysregulation plays a critical role in the pulmonary vascular and interstitial changes underlying PH [3]. This is best exemplified by the increased risk of developing pulmonary arterial hypertension (PAH) among patients with systemic autoimmune diseases, like systemic sclerosis (SSc) and systemic lupus erythematosus (SLE), which are grouped as WHO type 1 PH and subclassified as connective tissue disease (CTD)-PAH [4]. Importantly, immune involvement extends to idiopathic PAH (IPAH). Different immunological pathways that initiate or maintain the vasculopathy and vascular remodeling of IPAH have been elucidated [3,5,6,7,8,9,10,11,12,13,14]. Polarization of innate immune cells into pro-fibrotic subtypes, peri-vascular inflammation with extensive lymphocyte involvement, and association with autoimmunity by the presence of auto-antibodies against endothelial cells (AECAs) that influence vascular stromal cells contribute to endothelial activation and may propagate vascular remodeling in PAH [3,8,9,10,11,12,15,16,17,18]. This can result in disrupted endothelial-parenchymal crosstalk, characterized by elevated secretion of cytokines and chemokines such as TGF-β (transforming growth factor-β) and IL-8 (interleukin-8), dysregulated BMPR-2 (bone morphogenetic protein receptor-2) signaling, and the recruitment of circulating progenitor cells, including circulating endothelial colony-forming cells, which can exacerbate fibrosis in the pulmonary parenchyma [19,20,21,22]. Additionally, similar vascular changes in the bronchial vasculature might also contribute to inflammation and fibrosis. However, whether these immune-mediated vascular changes precede or contribute to the development of interstitial lung disease (ILD) remains under investigation and necessitates further research in future ILD studies [23].

Although PH is associated with connective tissue diseases (CTDs), it is rarely observed in idiopathic inflammatory myopathy (IIM), a condition characterized by myositis, ILD, and myositis-specific/associated antibodies (MSAs/MAAs) [24,25,26,27,28,29]. The prevalence of MSAs/MAAs in PH patients remains unclear, with reported rates varying widely. For instance, the prevalence of anti-synthetase antibodies can range from 8% to as high as 29% [30,31]. However, it is important to note that methodological differences in studies can significantly impact this reported percentage. When PH occurs in patients with IIM, it is often accompanied by extensive end-stage ILD [32]. The presence of PAH in patients with IIM without ILD is very rare. The involvement of immune-mediated vascular remodeling is likely to contribute to the pathogenesis, but much remains unknown due to its rarity. Vice versa, a large PAH cohort study screened 5223 patients for IIM, which yielded only 34 cases. Most of these had severe ILD, with only three patients having isolated PAH [33]. PH is currently not included in the diagnostic criteria of IIM, although mild PH may occur during more severe stages of ILD, classified under WHO type 3 [25,26,31,33,34,35,36].

In our expertise referral clinic, we questioned whether MSAs/MAAs are prevalent in patients presenting with PH but who had not been recognized as patients with an underlying systemic autoimmune disease and who had not been treated as such. We analyzed a cohort of pre-capillary and combined pre- and post-capillary PH patients for the presence of MSAs/MAAs. Because PH can develop in patients after or alongside pulmonary interstitial changes, often diagnosed as ILD, we also stratified our cohort based on the presence of pulmonary interstitial changes on radiological imaging suggestive of ILD [32]. We hypothesize that MSAs/MAAs are more prevalent in PH than currently suggested, especially in PH patients with ILD. 

## 2. Materials and Methods

### 2.1. Patient Selection

A retrospective cohort study was performed on adult patients who were diagnosed with PH in the past 20 years at the Maastricht University Medical Center (MUMC+) according to the diagnostic criteria at the time, with a mPAP ≥ 25 mmHg and a PVR ≥ 3 Wood Units assessed by RHC. Patients with combined pre- and post-capillary PH were also included. Patients with congenital heart disease, pulmonary veno-occlusive disease, and porto-pulmonary hypertension were excluded. Patients who were already diagnosed with an IIM were also excluded. PH patients underwent clinical evaluation by a clinical immunologist, who performed immunological diagnostics accordingly to evaluate the presence of a systemic immunological disease. Clinical characteristics were assessed and analyzed at the time of diagnosis of PH, including WHO type of PH, New York Heart Association (NYHA) class, hemodynamics measured by RHC, pulmonary function tests, a 6 min walking test (6MWT), and laboratory parameters, like N-terminal-pro-brain natriuretic peptide (NT-pro-BNP) and C-reactive protein (CRP). To determine the underlying cause of pulmonary hypertension (PH), all patients underwent high-resolution computed tomography (HRCT) as part of the diagnostic workup. Patients were stratified into the PH with ILD group when signs of pulmonary interstitial changes were present. To compare the prevalence observed in our PH cohort, routine clinical data were collected from patients suspected to have IIM (*n* = 558) and, hence, were tested for MSA/MAA. Obviously, in these patients, a diagnosis of IIM might be made or rejected. The clinical suspicion of IIM in these patients was established based on signs of myositis, like elevated creatine kinase (CK), proximal muscle weakness, muscle biopsy, electromyography, or typical skin lesions, with or without the presence of ILD. This study complies with the Declaration of Helsinki and was performed according to the local ethics committee’s approval.

### 2.2. Determination of Antibodies

Antibodies were detected in serum using a line-blot assay (EUROLINE Autoimmune Inflammatory Myopathies, EUROIMMUN, Lubeck, Germany). The assay identified MSAs against Jo-1, EJ, OJ, PL-7, PL-12 (anti-synthetase antibodies), Mi-2α, Mi-2β, TIF1-γ, MDA5, NXP2, and SAE1 (dermatomyositis antibodies), SRP (immune-mediated necrotizing myositis), and MAAs against Ku, PM/Scl-75, and PM/Scl-100 (overlap syndrome antibodies). A patient was considered positive for Mi-2 and PM/Scl antibodies if they tested positive for any subtype. In cases of both positive and weak positive results, the highest result was used to determine overall positivity. Data on Ro52 reactivity was excluded from our analysis. The immunoblot strips were analyzed with the EUROLINE Scan software version 3.4.34 (EUROIMMUN, Lubeck, Germany) according to the manufacturer’s recommendations for the EUROLINE Autoimmune Inflammatory Myopathies line blot assay. Strips were scored as negative, weak positive and positive, which corresponds with intensity levels of, respectively, 0–10, 11–25, and >25 [37]. Antibody reactivity on a positive and weak positive intensity level was separately evaluated. A patient is considered positive for MSAs/MAAs if at least one antibody reveals a reactivity >25; or weak positive if at least one antibody reveals a reactivity of 11–25 but none >25; all other patients are considered negative. The prevalence of MSA/MAA in our comparative cohort, as well as in healthy cohorts from the literature, was used for comparison [27,38,39]. 

### 2.3. Statistical Analysis

Categorical characteristics were expressed as numbers and percentages. Numerical data were expressed as mean and standard deviation (SD) or median and interquartile range (IQR) based on the normal distribution estimated by the Shapiro–Wilk test and data visualization. Statistics were performed using the Chi-Square and Fisher’s exact tests, respectively. A univariable and multivariable Cox proportional hazards regression analysis was performed for mortality risk. The statistical analysis was performed using IBM SPSS version 24.0 software. A *p*-value less than 0.05 was considered statistically significant. Figures were made with R version 4.0 and GraphPad Prism version 9.5.0.

## 3. Results

### 3.1. Study Population 

**Figure 1:**
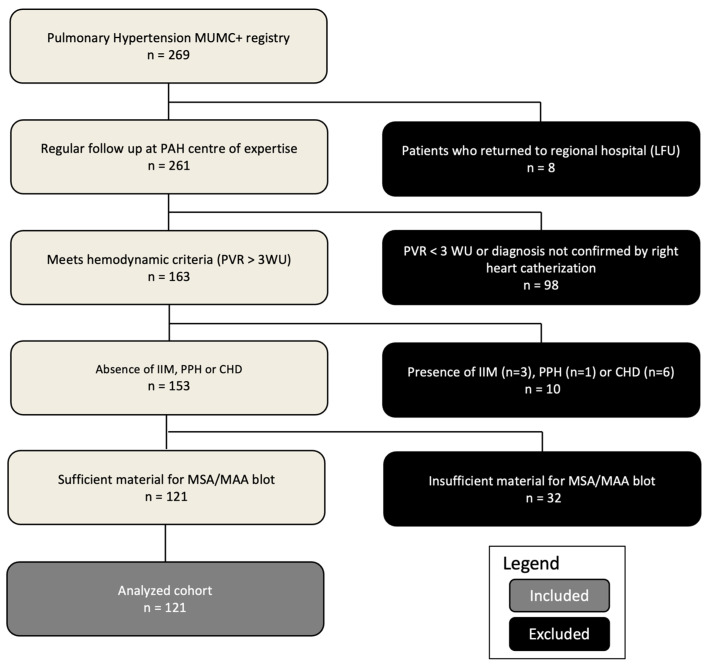
Flow chart for inclusion and exclusion of PH patients. PAH, pulmonary arterial hypertension; LFU, lost to follow-up; PVR, pulmonary vascular resistance; WU, wood units; IIM, idiopathic inflammatory myopathy; PPH, porto-pulmonary hypertension; CHD, congenital heart disease; MSA, myositis-specific antibodies; MAA, myositis-associated antibodies.

This study analyzed data from 269 patients with pulmonary hypertension (PH) registered in the MUMC+ PH database. Of these, 121 patients met the inclusion criteria and underwent myositis blot testing (Figure 1). The study cohort (*n* = 121) had a median age of 68 years (61.5–73.0) and was 57.9% female. Patients were classified according to the World Health Organization (WHO) system: 55 (45.5%) as WHO type 1 PAH, 9 (7.4%) as WHO type 2 (underlying heart disease with significant pre-capillary PH component), 35 (28.9%) as WHO type 3 (underlying pulmonary disease), 18 (14.9%) as WHO type 4 CTEPH, and 4 (3.3%) as WHO type 5 (multifactorial etiologies). The median follow-up duration was 4.2 years (2.2–6.0). Baseline characteristics for this cohort are summarized in Table 1. 

### 3.2. Cardiopulmonary Characteristics 

Baseline hemodynamics confirmed PH, with a mean mPAP of 40.1 ± 9.4 mmHg. Median PAWP was 12 mmHg (8–14.5 mmHg), and median PVR was 425 dynes/sec/cm^−5^ (342–576 dynes/sec/cm^−5^). Exercise capacity was impaired, with a mean 6 min walk distance (6 MWD) of 330 ± 130 m. Most patients (57%) were in NYHA functional class III/IV. Table 1 provides additional cardiac and hemodynamic data. Pulmonary function testing revealed a mean forced expiratory volume in one second (FEV1) of 74.8% ± 20.4% predicted, a mean forced vital capacity (FVC) of 86.9% ± 16.8% predicted, and a mean total lung capacity (TLC) of 87.0% ± 18.3% predicted. The median diffusing capacity of the lungs for carbon monoxide (KCO) was reduced at 55% predicted (41–74%). Significant differences in TLC and KCO were observed between PH patients with and without ILD on imaging (*p* = 0.003, *p* < 0.001, Table 1). Radiological imaging revealed interstitial changes in 35 patients (38.9%). The distribution of HRCT ILD patterns is shown in Table 2. Systemic sclerosis was five times more prevalent in the PH with ILD group (*p* = 0.005).

### 3.3. Prevalence of Myositis-Specific Antibodies (MSA) and Myositis-Associated Antibodies (MAA) in PH Patients

**Figure 2:**
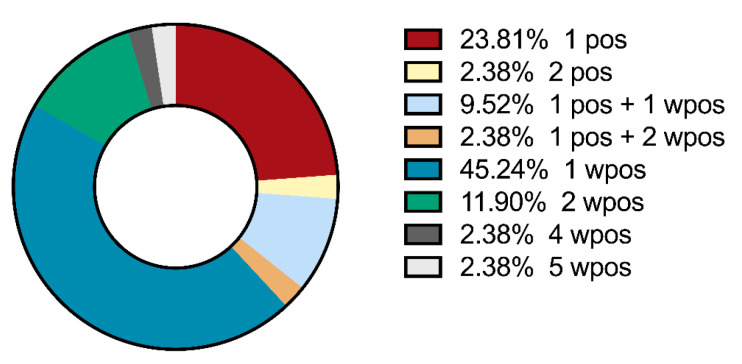
Distribution of single and multiple (weak) positivities of MSAs and MAAs in PH patients (*n* = 121).

In the PH cohort (*n* = 121), 16 patients (13.2%) had at least one positive MSA/MAA, while 26 patients (21.5%) had no positive but at least one weak positive MSA/MAA (Table 3). In this cohort, in total, 17 positive and 44 weak positive reactivities were observed (Table 4). The distribution of single and multiple MSA/MAA (weak) positivity is depicted in Figure 2.

**Figure 3:**
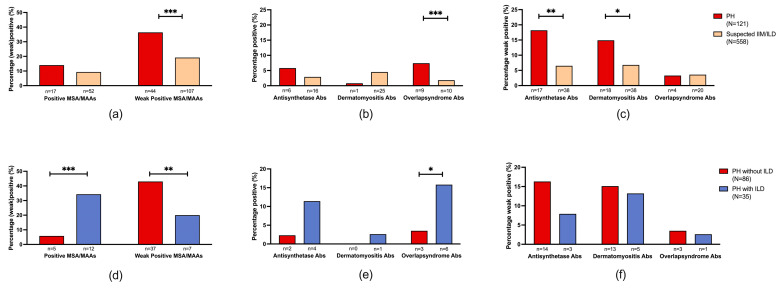
Percentages of positive and weak positive antibodies with MSA/MAA grouped by association with disease in the PH with and without ILD and the suspected IIM/ILD cohort. (**a**) Total positive and weak positive MSA/MAA in the PH cohort (*n* = 121) and suspected IIM/ILD patients (*n* = 558). (**b**) positive MSA/MAA in PH and suspected IIM/ILD patients. (**c**) weak positive MSA/MAA in PH and suspected IIM/ILD patients. (**d**) Total positive and weak positive MSA/MAA in PH with (*n* = 81)/without (*n* = 35) ILD patients. (**e**) positive MSA/MAA in PH with/without ILD patients (**f**) weak positive MSA/MAA in PH with/without IL patients. MSA/MAA were grouped as follows: anti-synthetase (Jo1, OJ, PL7, PL12, EJ), dermatomyositis (Mi2, NXP2, SAE1, MDA5, Tif1y), and CTD overlap syndrome (Ku, Pm/Scl). Significance is depicted with * (<0.05), ** (<0.02), and *** (<0.001).

We compared total MSA/MAA prevalence, grouped by associated clinical myositis syndromes, to a cohort of suspected IIM/ILD patients. This revealed a significantly higher prevalence of total weak positive (36.4%, *n*/*N* = 44/121) MSAs/MAAs in the PH cohort when compared with weak positive MSAs/MAAs (19.2%, *n*/*N* = 107/558, *p* < 0.001) in the suspected IIM/ILD cohort (Figure 3a). No significant difference was observed when the prevalence of positive MSAs/MAAs in the PH cohort (14.0%, *n*/*N* = 17/121) was compared with the suspected IIM/ILD cohort (9.3%, *n*/*N* = 52/558, *p* = 0.222) (Figure 3a).

Specifically, the PH cohort did not demonstrate a significant difference in the prevalence of positive anti-synthetase syndrome-associated MSAs (5.0%, *n*/*N* = 6/121 vs. 2.9%, *n*/*N* = 16/558, *p* = 0.25) in suspected IIM/ILD. However, weak positive anti-synthetase syndrome-associated MSAs were significantly more prevalent in the PH cohort (14.0%, *n*/*N* = 17/121 vs. 6.8%, *n*/*N* = 38/558 in suspected IIM/ILD, *p* = 0.008) (Figure 3b,c). Positive dermatomyositis-associated MSAs were less prevalent in the PH cohort (0.8%, *n*/*N* = 1/121) compared to the suspected IIM/ILD cohort (4.5%, *n*/*N* = 25/558, *p* = 0.058). However, weak positive dermatomyositis-associated MSAs/MAAs were more prevalent in the PH cohort (14.9%, *n*/*N* = 18/121 vs. 6.8%, *n*/*N* = 38/558 in suspected IIM/ILD, *p* = 0.011) (Figure 3b,c). The prevalence of positive overlap syndrome-associated MSAs/MAAs was significantly higher in the PH cohort (7.4%, *n*/*N* = 9/121) in comparison with the suspected IIM/ILD cohort (1.8%, *n*/*N* = 10/558, *p* < 0.001). No significant difference was observed in the prevalence of weak positive overlap syndrome-associated MSAs/MAAs (3.3%, *n*/*N* = 4/121 in PH vs. 3.6%, *n*/*N*= 20/558 in suspected IIM/ILD, *p* = 0.783) (Figure 3b,c).

### 3.4. Prevalence of Myositis-Specific Antibodies (MSA) and Myositis-Associated Antibodies (MAA) in PH Patients with or without Radiological Signs of ILD

We stratified the PH cohort based on the presence or absence of interstitial changes on imaging and MSA/MAA findings (Table 3). The PH with ILD group had a significantly higher prevalence of patients with at least one positive MSA/MAA (*n*/*N* = 11/35; 31.4%) compared to the PH without ILD group (*n*/*N* = 5/86; 5.8%, *p* < 0.001). The prevalence of patients with >1 weak positive but no positive MSA/MAA did not differ significantly between the two groups. See Table 4 and the Appendix A for multi-antibody positivity, distribution, antigen specificity, and antibody prevalence per patient (Table A1).

The total prevalence of positive MSAs/MAAs was significantly higher in the PH with ILD cohort (34.3%, *n*/*N* = 12/35) compared to the PH without ILD cohort (5.8%, *n*/*N* = 5/86, *p* < 0.001) (Figure 3d). This was also significantly higher when compared to the suspected IIM/ILD cohort (9.3%, *n*/*N* = 52/558, *p* < 0.001). The total prevalence of weak positive MSAs/MAAs was significantly higher in the PH without ILD cohort (43.0%, *n*/*N* = 37/86) compared to the PH with ILD cohort (20.0%, *n*/*N* = 7/35, *p* = 0.005) (Figure 3d).

No significant difference was observed when the prevalence of positive anti-synthetase syndrome-associated MSAs was compared between the PH with and without ILD patients (*p* = 0.057). Positive overlap syndrome-associated MAAs were significantly more prevalent in the PH with ILD cohort (17.1%, *n*/*N* = 6/35) compared to the PH without ILD cohort (3.5%, *n*/*N* = 3/86, *p* = 0.017) (Figure 3e). No significant difference was observed when the prevalence of weak positive anti-synthetase syndrome-associated (*p* = 0.389), dermatomyositis-associated (*p* = 1.000), and overlap syndrome-associated antibodies (*p* = 0.497) were compared between PH patients with or without ILD.

### 3.5. Multivariable Cox Proportional Hazards Regression Analysis for Mortality Risk

We aimed to explore whether MSA/MAA (weak) positivity with or without ILD affected survival outcomes. However, no significant differences in survival between these subgroups were observed (*p* = 0.220) (Figure A2). After univariable analyses, a multivariable Cox proportional hazards regression analysis was performed to identify factors associated with the risk of death in patients with PAH. Model 1 included age, BMI, sex, MSA/MAA positivity, and ILD as predictor variables (Table 5). Higher age (+5 years, HR = 1.42 [95%CI: 1.24–1.66]; *p* < 0.001) and the presence of ILD (HR = 2.02 [95%CI: 1.15–3.56]; *p* = 0.015) were independently associated with mortality risk. A backward selection process revealed age (+5 years, HR = 1.42 [95%CI: 1.24–1.65]; *p* < 0.001) and ILD (HR = 2.17 [95%CI: 1.28–3.68]; *p* = 0.004) as the strongest predictors for death in PAH. The overall model performance was statistically significant (χ^2^ = 31.29, df = 2; *p* < 0.001) with a C-index of 0.716, indicating sufficient discrimination. Thus, the addition of BMI, sex, and MSA/MAA positivity to the prognostic model did not demonstrate a significantly better fit for predicting the risk of death in PAH as compared to model 2 (*p* = 0.105).

## 4. Discussion

This is the first study to systematically assess MSA/MAA prevalence in a large PH cohort. We observed a significantly higher prevalence of positive MSAs/MAAs in PH patients with radiological signs of ILD and weak positive MSAs/MAAs in PH patients without radiological signs of ILD compared to a cohort with suspected IIM/ILD, who were specifically tested for the presence of these autoantibodies. Notably, MSAs/MAAs were detected in PH patients both with and without interstitial abnormalities on HRCT, although positivity tended to be weaker in the absence of radiographic ILD. We employed established nomenclature for clinical associations, with anti-synthetase and anti-overlap syndrome antibodies being the most frequently detected [24]. Importantly, patients in this study were referred to our PH clinic without prior diagnosis or treatment for immune-mediated inflammatory myopathies, or ILD, and lacked signs of myositis at presentation. These findings suggest that PH should be considered within the diagnostic criteria for MSA/MAA-associated diseases.

Previous studies report varying MSA/MAA prevalence in healthy controls. Ghirardello et al. showed a prevalence of 28.6% of MSA/MAA in healthy controls [27]. However, Ro52 was included and was positive in 73.4% of the MSA/MAA positive cases. Besides, assay results were positive ≥11 AU. Vulsteke et al. showed MSA/MAA positivity of 3/40 (7.5%) in blood donors; however, cut-off values were not mentioned [38]. Espinosa et al. showed MSA/MAA positivity of 3/60 (5%) in healthy controls; however, cut-off values of ≥11 AU were also used [39]. Prior studies in IIM or ILD patients demonstrate a variable MSA/MAA prevalence. One study showed a prevalence of 27% MSA/MAA positivity in ILD patients. However, 36% of these patients were anti-Ro52-positive [28]. Others showed a prevalence of MSAs/MAAs in IIM of around 50% [38]. Remarkably, the cut-off value for the positivity of MSAs/MAAs was ≥11 AU in that study. Our study employed stricter criteria, in which we excluded Ro52 and used a higher cut-off of ≥26 AU, resulting in a potentially more specific assessment of MSA/MAA positivity. This lack of standardization complicates direct comparisons with our findings. To address this, we compared our PH cohort to MSA/MAA line blot results from patients suspected of IIM (*n* = 558) tested at our hospital. This internal control group allows for meaningful comparisons, as the same assay and criteria were used. The significantly higher prevalence of MSAs/MAAs in our PH cohort suggests an association between these antibodies and PH.

**Figure A1:**
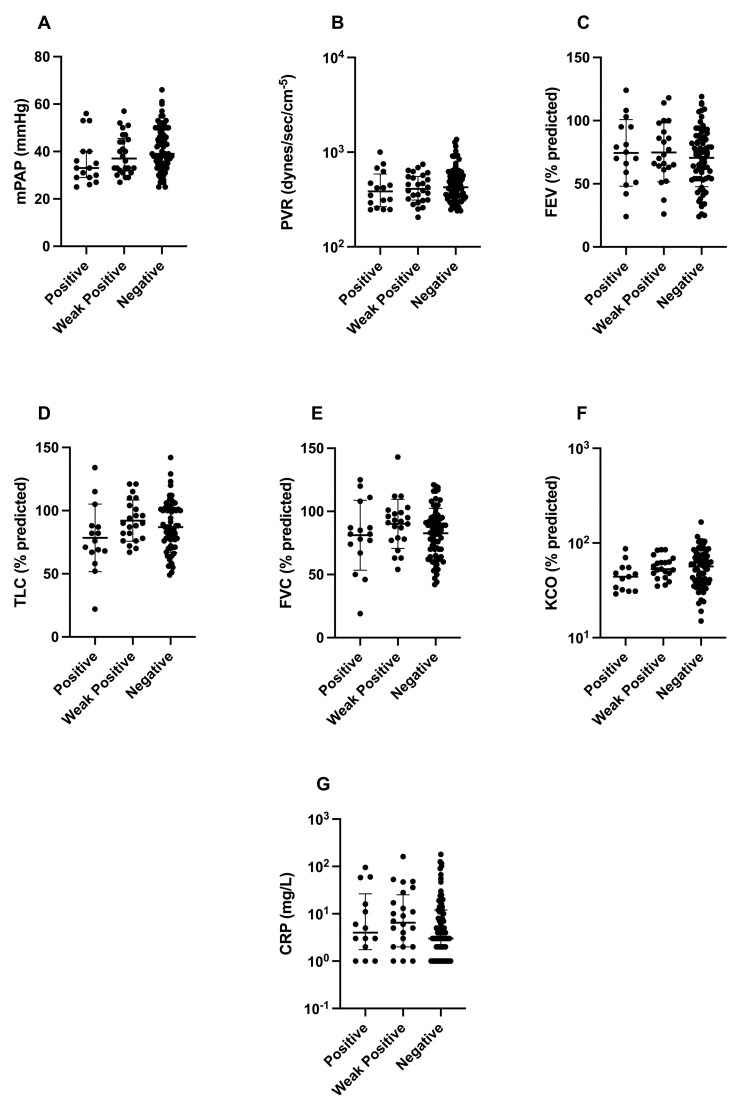
Cardiopulmonary hemodynamic and pulmonary functional measurements and 5-year survival in PH patients with positive, weak positive, and negative MSAs/MAAs. (**A**) mean pulmonary arterial pressure in mmHg, (**B**) pulmonary vascular resistance in dynes/sec/cm^−5^, (**C**) forced expiratory volume as percentage of predicted, (**D**) total lung capacity as percentage of predicted, (**E**) forced vital capacity as percentage of predicted, (**F**) carbon oxide transfer coefficient as percentage of predicted, (**G**) C-reactive protein in mg/L. Horizontal bars represent either median values with an interquartile range or mean values with a standard deviation according to the normality test. Statistical analysis was performed using the Kruskal–Wallis and Mann–Whitney tests.

**Figure A2:**
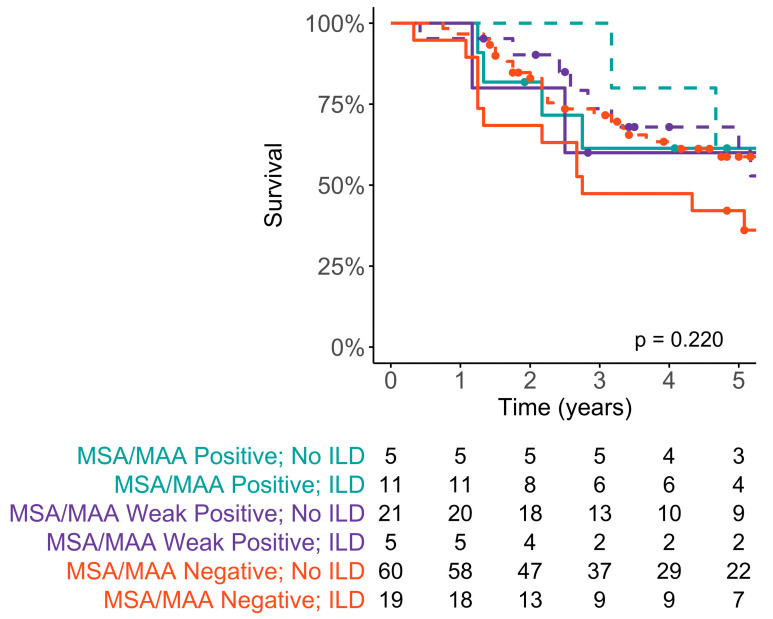
Kaplan–Meyer subgroup survival analysis of PH patients with/without ILD or MSA/MAA (weak) positivity. Subgroups without ILD are depicted with a dotted line. Statistical analysis was performed using the log-rank test.

The association between IIM, anti-synthetase syndrome, and PH has been established in the literature before [26,31,33,34,40]. However, in most cases, a diagnosis of active myositis and often severe ILD was found. Patients in our cohort had no signs of active myositis or severe end-stage ILD. Although the prevalence of MSAs/MAAs was highest in the PH with ILD group, most patients in our cohort positive for MSAs/MAAs showed, to some extent, interstitial pulmonary disease on radiographical imaging. However, these patients often did not present with a classified ILD diagnosis prior to a PH diagnosis. Interestingly, even the patients without interstitial pulmonary involvement did also show weak positivity for MSAs/MAAs. If the cut-off values used in other studies were applied, these patients would have been classified as positive. When we compared pulmonary voluminal measurements between patients with positive MSAs/MAAs and patients with weak antibody positivity, no significant difference was observed (Figure A1). Moreover, since the ILD observed was classified as only moderately mild based on pulmonary functional testing, the elevated pulmonary arterial pressure could not be explained directly by interstitial changes alone [41]. This is also known as out-of-proportion PAH, which could suggest that PH in these patients might have developed due to immune-mediated vascular remodeling. Notably, our findings suggest that in PH patients with radiological signs of ILD who tested positive for MSA/MAA, a retrospective diagnosis of IIM should be considered even without overt clinical myositis. This highlights the importance of screening for MSA/MAA in PH, especially in the presence of mild radiological signs of ILD, as IIM prevalence may be underestimated in this population. Subgroup survival analysis of our study population did not reveal any significant differences. However, multivariate Cox regression indicated that higher age at the time of diagnosis and the presence of signs of ILD on radiological imaging were independently correlated with an increased mortality risk. This association was not observed for MSA/MAA (weak) positivity. Nonetheless, since MSA/MAA presence is strongly correlated with ILD or its potential development over the course of the disease, it also underscores the importance of screening for MSA/MAA in patients with PH. This study has limitations inherent to its retrospective design, including potential selection bias and confounding factors. The single-center nature limits generalizability. This study opted for a disease-control group rather than a healthy-control group due to concerns that MSA/MAA levels detected in healthy individuals with no apparent illness might partially reflect assay limitations or subclinical immune processes. The ILD diagnosis in this study relied on HRCT evaluation by different radiologists. This approach may introduce bias since HRCT interpretation can be subjective, particularly for subtle or early stage ILD.

Until now, PH associated with MSAs/MAAs has been merely interpreted as a complication of severe or end-stage ILD in IIM patients [34]. However, this study provides new immunological insights into pre-capillary and combined pre-post capillary PH associated with MSAs/MAAs, showing a high prevalence of (weak) positive MSAs/MAAs with and without interstitial pulmonary disease. The progressive nature of immune-mediated diseases, with their shifting pathophysiological mechanisms, underscores the importance of early recognition of autoimmunity in PH. PH patients with lower MSA/MAA titers without overt interstitial changes may represent an earlier disease stage. We propose a case series with longitudinal follow-up of pulmonary function and imaging in patients presenting with (weak) MSA/MAA positivity and PH. This study could elucidate disease progression and further investigate the role of immunosuppressive treatment in PH. Based on our findings, we strongly advocate for routine MSA/MAA screening in newly diagnosed PH patients, especially in those with (mild) radiological signs of ILD. Integrating MSA/MAA positivity into screening tools, such as the one validated by Parikh et al. [42,43] for PH in ILD, could enhance early detection. Future research should also focus on elucidating the fundamental immunological mechanisms associated with MSAs/MAAs and their role in vascular remodeling in pre-capillary and combined pre- and post-capillary PH patients. We propose substantial immune profiling of MSA/MAA (weak) positive patients to investigate whether the presence of these antibodies can help to distinguish immune-mediated PH patients. Further investigation should focus on vascular remodeling-inducing properties in biohybrid assays. Investigation of the interaction of vascular stromal cells and serum or isolated immunoglobulins obtained from PH patients with signs of autoimmunity, like MSA/MAA presence, could help to substantiate the role of immunologically targeted therapy that could prevent further progression of immune-mediated vascular remodeling into end-stage PH.

## Figures and Tables

**Table 1:** Demographic, clinical characteristics, and laboratory features of the study population.

	PH Patients (*n* = 121)	PH without ILD (*n* = 86)	PH with ILD (*n* = 35)	*p*-Value
Gender, female (%)	70 (57.9%)	56 (65.1%)	14 (40.0%)	0.011
Age, y	68.0 (61.5; 73.0)	69.0 (62.0; 72.8)	68.0 (59.0; 74.0)	1.000
BMI, kg/m^2^	28.7 (24.3; 33.2)	29.9 (24.7; 34.1)	26.3 (23.1; 28.7)	0.002
Total follow up time, y	4.2 (2.2; 6.0)	4.1 (2.3; 6.4)	2.8 (1.3; 5.8)	0.208
NYHA I-II (%)	52 (43.0%)	39 (45.3%)	13 (37.1%)	0.408
NYHA III-IV (%)	69 (57.0%)	47 (54.7%)	22 (62.9%)	0.408
6MWT, m	330 ± 130	337 ± 125	299 ± 131	0.429
NT-pro BNP, pmol/L	163 (41; 340)	132 (40; 315)	184 (82; 449)	0.498
CRP, mg/L	3 (2; 11)	3 (2; 9)	4 (2; 20)	0.408
Baseline right heart catheterization				
mPAP, mmHg	40.1 ± 9.4	40.5 ± 10.1	39.1 ± 8.9	0.530
Capillary wedge pressure, mmHg	12.0 (8.0; 14.5)	12 (7.3; 14.8)	10 (8.0; 13.0)	0.857
PVR, dynes/sec/cm^−5^	425 (342; 576)	432 (338; 579)	421 (340; 622)	0.981
Baseline pulmonary function				
FEV%	74.8 ± 20.4	70.8 ± 20.9	78.3 ± 21.1	0.050
FVC%	86.9 ± 16.8	85.5 ± 17.9	85.8 ± 22.8	0.567
TLC%	87.0 ± 18.3	90.3 ± 18.5	77.5 ± 20.7	0.003
KCO%	55.0 (41; 74)	66.0 (48; 83)	42.0 (33; 49)	<0.001
WHO classification (1–5)	55/9/35/18/4	39/8/19/18/2	16/1/16/0/2	
Connective tissue disease		10	15	<0.001
Systemic Sclerosis	12	4	8	0.005
Systemic Lupus Erythematosus	4	2	2	0.578
MCTD	4	1	3	0.072
Rheumatoid arthritis	2	2	0	1.000
Sjogren syndrome	1	1	0	1.000
Dermatomyositis	1	0	1	0.289
Sarcoidosis	1	0	1	0.289

Count (percentage), mean ± standard deviation, or median (interquartile range) were given, as appropriate. *p*-values were calculated using the Chi-square/Fisher’s exact test, analysis of variance, or Kruskal–Wallis test, respectively. ILD, interstitial lung disease (stratification based on radiological imaging); BMI, body mass index; NYHA, New York Heart Association; 6MWT, six-minute walking test; NT-pro BNP, N-terminal pro-brain natriuretic peptide; CRP, C-reactive protein; mPAP, mean pulmonary arterial pressure; PVR, pulmonary vascular resistance; FEV%, forced expiratory volume %; FVC%, forced vital capacity %; TLC%, total lung capacity %; KCO, corrected carbon mono-oxide transfer coefficient; WHO, world health organization; MCTD, mixed connective tissue disease.

**Table 2:** ILD patterns on HRCT in PH patients (*n* = 35).

ILD Pattern	(*n* = 35)
NSIP	10 (28.6%)
UIP	5 (14.3%)
RB-ILD	2 (5.7%)
CPFE	1 (2.9%)
Nodular	1 (2.9%)
Reticular	4 (11.4%)
Subpleural	3 (8.6%)
Aspecific	9 (25.7%)

ILD, interstitial lung disease; NSIP, non-specific interstitial pneumonia; UIP, usual interstitial pneumonia; RB-ILD, respiratory bronchiolitis-interstitial lung disease; CPFE, common pulmonary fibrosis and emphysema.

**Table 3:** Distribution of single and multiple (weak) positivities of MSAs and MAAs in PH patients with or without ILD.

Characteristics	PH Patients (*n* = 121)	PH without ILD (*n* = 86)	PH with ILD (*n* = 35)	*p*-Value
Negative	79 (65.3%)	60 (69.8%)	19 (54.3%)	0.105
≥1 positive antibody	16 (13.2%)	5 (5.8%)	11 (31.4%)	<0.001
≥1 weak positive antibody	26 (21.5%)	21 (24.4%)	5 (14.3%)	0.329

PH, pulmonary hypertension; ILD, interstitial lung disease.

**Table 4:** Prevalence of MMAs/MSAs in PH patients (*n* = 121).

MSA/MAA (*n* = 121)	Positive	Weak Positive
OJ	1 (0.8%)	2 (1.6%)
EJ	1 (0.8%)	0
PL-7	3 (2.5%)	11 (9.1%)
PL-12	0	3 (2.5%)
JO-1	1 (0.8%)	1 (0.8%)
SRP	1 (0.8%)	5 (4.1%)
SAE-1	0	4 (3.3%)
MDA-5	0	8 (6.6%)
NXP-2	0	0
Tif-1γ	0	1 (0.8%)
Mi-2	1 (0.8%)	5 (4.1%)
PM/SCL	6 (5.0%)	2 (1.6%)
Ku	3 (2.5%)	2 (1.6%)

MSA, myositis-specifis antibodies; MAA, myositis-associated antibodies.

**Table 5:** Hazard ratios after Cox proportional hazard regression for death in PAH.

	Univariable			Multivariable Model 1 (*C*-index = 0.752)			Multivariable Model 2 (*C*-index = 0.716)		
Predictors	HR	95% CI	*p*-Value	HR	95% CI	*p*-Value	HR	95% CI	*p*-Value
Age [+5 years]	1.37	1.20–1.57	<0.001	1.43	1.24–1.66	<0.001	1.42	1.24–1.65	<0.001
BMI [+1 kg/m^2^]	0.97	0.93–1.01	0.093	0.99	0.94–1.03	0.545	-	-	-
Sex [Male]	1.82	1.11–2.99	0.018	1.65	0.95–2.84	0.074	-	-	-
MSA/MAA [Weak Positive]	0.82	0.43–1.57	0.548	0.78	0.41–1.50	0.455	-	-	-
MSA/MAA [Positive]	1.00	0.50–1.99	0.990	0.58	0.28–1.22	0.152	-	-	-
ILD [Yes]	1.74	1.05–2.90	0.033	2.02	1.15–3.56	0.015	2.17	1.28–3.68	0.004

## Data Availability

This article contains all the data used for this article.

## Appendix A

**Table A1:** Clinical characteristics and MSA/MAA positivity per patient.

Patient ID	Sex m/f	Age at Inclusion	PH WHO Type	Radiological Signs of ILD y/n	MMA/MSA
1	0	64	1	y	neg
2	0	73	4	n	wpos Jo1
3	1	56	5	y	pos PM-Scl
4	0	54	1	y	pos PM-Scl
5	0	59	3	y	neg
6	0	77	2	n	pos EJ
7	0	65	1	y	neg
8	0	59	1	y	pos Jo1
9	1	82	2	n	neg
10	1	78	3	y	pos PM-Scl
12	0	75	3	y	neg
13	1	56	1	y	neg
15	1	77	3	y	neg
17	1	67	1	n	neg
18	1	53	1	n	neg
20	1	60	5	y	wpos OJ, wpos Mi2
21	0	44	1	n	neg
23	0	79	1	n	neg
24	0	61	1	n	neg
26	0	66	4	n	neg
27	0	71	1	n	wpos PL7 wpos MDA5
29	0	66	2	n	wpos PL7
30	0	69	4	n	wpos MDA5
31	0	63	1	y	neg
32	1	70	3	n	neg
33	0	75	3	n	neg
37	1	73	4	n	neg
38	1	65	3	y	neg
39	0	75	2	n	wpos SRP wpos PL12 wpos Mi2 wpos MDA5 wpos Ku
40	0	69	4	n	pos PM-Scl wpos Mi2
41	1	75	4	n	wpos PL7
43	0	68	1	y	pos Ku wpos SAE1 wpos MDA5
46	0	66	1	n	neg
47	0	60	1	n	neg
50	0	59	3	n	wpos SRP
52	0	74	1	n	neg
53	0	69	1	n	neg
54	1	57	5	n	wpos MDA5
56	1	67	1	y	neg
57	0	74	1	y	pos PM-Scl
64	0	59	1	n	neg
66	0	73	3	y	pos OJ,
67	0	80	2	y	wpos Mi2
69	1	66	3	n	neg
70	0	62	5	n	neg
73	0	72	1	n	wpos MDA5
78	1	40	1	n	neg
81	1	75	3	y	neg
83	0	64	4	n	neg
85	1	80	4	n	neg
86	0	65	3	n	neg
87	1	60	1	y	neg
88	0	75	1	n	neg
91	0	63	3	n	neg
93	1	68	3	y	pos SRP
94	0	75	2	n	wpos SRP wpos PL7
95	1	77	3	n	wpos OJ
103	0	79	1	n	wpos PL7
104	0	56	4	n	neg
106	0	64	1	n	neg
107	1	79	3	n	pos Ku wpos PL7
108	1	50	1	y	wpos Ku
111	0	47	1	n	neg
119	0	70	4	n	wpos PL7 wpos PM-Scl
120	1	46	1	n	wpos PL7
124	1	72	2	n	neg
126	1	39	1	y	neg
127	0	87	2	n	pos PL7 wpos PM-Scl
134	0	66	1	n	wpos MDA5
141	0	66	4	n	neg
162	1	89	3	n	neg
164	1	23	4	n	neg
165	0	71	1	n	pos PM-Scl
168	1	76	3	y	neg
175	1	68	3	y	neg
181	0	81	4	n	neg
186	1	71	3	y	neg
191	0	73	1	n	neg
194	0	63	1	n	neg
195	1	72	1	y	neg
197	0	46	1	n	neg
198	0	66	1	n	neg
202	0	27	1	n	neg
205	0	71	1	n	neg
208	0	59	3	y	neg
213	0	63	1	n	neg
217	0	70	3	n	neg
220	1	75	3	y	neg
223	0	71	3	n	neg
228	0	70	3	n	neg
233	0	70	3	n	neg
246	0	64	1	y	pos PL7 pos Ku
250	1	72	1	n	neg
254	1	71	4	n	neg
271	1	74	3	n	neg
273	0	46	1	n	wpos SAE1
279	1	57	1	n	neg
285	0	74	3	n	neg
293	1	78	3	n	neg
301	1	72	3	y	wpos PL7
306	1	66	4	n	neg
309	1	55	2	n	neg
310	1	73	1	y	pos Mi2, wpos PL12
311	0	71	1	n	neg
312	0	66	4	n	wpos PL7
313	1	74	1	n	neg
314	1	75	3	y	neg
315	0	49	3	n	neg
316	1	57	1	n	wpos Tif1y
317	1	68	3	y	wpos SAE1 wpos MDA5
318	0	69	4	n	wpos SAE1
319	1	72	1	n	wpos
320	0	63	1	n	neg
321	0	50	1	y	pos PL7
322	1	73	1	n	neg
323	1	57	1	n	neg
324	0	64	3	n	neg
325	0	53	1	n	neg
326	1	65	4	n	neg
327	0	62	1	n	wpos SRP wpos PL7 wpos PL12 wpos Mi-2
328	1	69	3	n	neg

Survival analysis of myositis-specific antibodies (MSA) and myositis-associated antibodies (MAA) (weak) positive PH patients with and without ILD.

We aimed to explore whether MSA/MAA (weak) positivity with or without ILD affected survival outcomes. However, no significant differences in survival between these subgroups were observed (*p* = 0.220) (Figure A2).

## References

[1] Chang K.Y., Duval S., Badesch D.B., Bull T.M., Chakinala M.M., De Marco T., Frantz R.P., Hemnes A., Mathai S.C., Rosenzweig E.B. (2022). Mortality in Pulmonary Arterial Hypertension in the Modern Era: Early Insights From the Pulmonary Hypertension Association Registry. J. Am. Heart Assoc..

[2] Humbert M., Kovacs G., Hoeper M.M., Badagliacca R., Berger R.M.F., Brida M., Carlsen J., Coats A.J.S., Escribano-Subias P., Ferrari P. (2023). 2022 ESC/ERS Guidelines for the diagnosis and treatment of pulmonary hypertension. G. Ital. Cardiol..

[3] Tobal R., Potjewijd J., van Empel V.P.M., Ysermans R., Schurgers L.J., Reutelingsperger C.P., Damoiseaux J., van Paassen P. (2021). Vascular Remodeling in Pulmonary Arterial Hypertension: The Potential Involvement of Innate and Adaptive Immunity. Front. Med..

[4] Thoreau B., Mouthon L. (2023). Pulmonary arterial hypertension associated with connective tissue diseases (CTD-PAH): Recent and advanced data. Autoimmun. Rev..

[5] Dhala A. (2012). Pulmonary arterial hypertension in systemic lupus erythematosus: Current status and future direction. Clin. Dev. Immunol..

[6] Parperis K., Velidakis N., Khattab E., Gkougkoudi E., Kadoglou N.P.E. (2023). Systemic Lupus Erythematosus and Pulmonary Hypertension. Int. J. Mol. Sci..

[7] Naranjo M., Hassoun P.M. (2021). Systemic Sclerosis-Associated Pulmonary Hypertension: Spectrum and Impact. Diagnostics.

[8] Arends S.J., Damoiseaux J.G., Duijvestijn A.M., Debrus-Palmans L., Boomars K.A., Brunner-La Rocca H.P., Cohen Tervaert J.W., van Paassen P. (2013). Functional implications of IgG anti-endothelial cell antibodies in pulmonary arterial hypertension. Autoimmunity.

[9] Koudstaal T., Boomars K.A. (2023). Inflammatory biomarkers in pulmonary arterial hypertension: Ready for clinical implementation?. Eur. Respir. J..

[10] Koudstaal T., Boomars K.A., Kool M. (2020). Pulmonary Arterial Hypertension and Chronic Thromboembolic Pulmonary Hypertension: An Immunological Perspective. J. Clin. Med..

[11] Koudstaal T., van Uden D., van Hulst J.A.C., Heukels P., Bergen I.M., Geenen L.W., Baggen V.J.M., van den Bosch A.E., van den Toorn L.M., Chandoesing P.P. (2021). Plasma markers in pulmonary hypertension subgroups correlate with patient survival. Respir. Res..

[12] El Kasmi K.C., Pugliese S.C., Riddle S.R., Poth J.M., Anderson A.L., Frid M.G., Li M., Pullamsetti S.S., Savai R., Nagel M.A. (2014). Adventitial fibroblasts induce a distinct proinflammatory/profibrotic macrophage phenotype in pulmonary hypertension. J. Immunol..

[13] Heukels P., Corneth O.B.J., van Uden D., van Hulst J.A.C., van den Toorn L.M., van den Bosch A.E., Wijsenbeek M.S., Boomars K.A., Kool M., Hendriks R.W. (2021). Loss of immune homeostasis in patients with idiopathic pulmonary arterial hypertension. Thorax.

[14] Price L.C., Wort S.J., Perros F., Dorfmuller P., Huertas A., Montani D., Cohen-Kaminsky S., Humbert M. (2012). Inflammation in pulmonary arterial hypertension. Chest.

[15] Arends S.J., Damoiseaux J.G., Duijvestijn A.M., Debrus-Palmans L., Vroomen M., Boomars K.A., Brunner-La Rocca H.P., Reutelingsperger C.P., Cohen Tervaert J.W., van Paassen P. (2013). Immunoglobulin G anti-endothelial cell antibodies: Inducers of endothelial cell apoptosis in pulmonary arterial hypertension?. Clin. Exp. Immunol..

[16] Arends S.J., Damoiseaux J., Duijvestijn A., Debrus-Palmans L., Boomars K., Broers B., Tervaert J.W., van Paassen P. (2010). Prevalence of anti-endothelial cell antibodies in idiopathic pulmonary arterial hypertension. Eur. Respir. J..

[17] Stenmark K.R., Nozik-Grayck E., Gerasimovskaya E., Anwar A., Li M., Riddle S., Frid M. (2011). The adventitia: Essential role in pulmonary vascular remodeling. Compr. Physiol..

[18] Marsh L.M., Jandl K., Grunig G., Foris V., Bashir M., Ghanim B., Klepetko W., Olschewski H., Olschewski A., Kwapiszewska G. (2018). The inflammatory cell landscape in the lungs of patients with idiopathic pulmonary arterial hypertension. Eur. Respir. J..

[19] Borek I., Birnhuber A., Voelkel N.F., Marsh L.M., Kwapiszewska G. (2023). The vascular perspective on acute and chronic lung disease. J. Clin. Investig..

[20] Fliesser E., Lins T., Berg J.L., Kolb M., Kwapiszewska G. (2023). The endothelium in lung fibrosis: A core signaling hub in disease pathogenesis?. Am. J. Physiol. Cell Physiol..

[21] Yanagihara T., Tsubouchi K., Zhou Q., Chong M., Otsubo K., Isshiki T., Schupp J.C., Sato S., Scallan C., Upagupta C. (2023). Vascular-Parenchymal Cross-Talk Promotes Lung Fibrosis through BMPR2 Signaling. Am. J. Respir. Crit. Care Med..

[22] Billoir P., Blandinieres A., Gendron N., Chocron R., Gunther S., Philippe A., Guerin C.L., Israel-Biet D., Smadja D.M. (2021). Endothelial Colony-Forming Cells from Idiopathic Pulmonary Fibrosis Patients Have a High Procoagulant Potential. Stem Cell Rev. Rep..

[23] Johnson S.R., Granton J.T. (2011). Pulmonary hypertension in systemic sclerosis and systemic lupus erythematosus. Eur. Respir. Rev..

[24] Betteridge Z., McHugh N. (2016). Myositis-specific autoantibodies: An important tool to support diagnosis of myositis. J. Intern. Med..

[25] Damoiseaux J., Mammen A.L., Piette Y., Benveniste O., Allenbach Y., ENMC 256th Workshop Study Group (2022). 256(th) ENMC international workshop: Myositis specific and associated autoantibodies (MSA-ab): Amsterdam, The Netherlands, 8–10 October 2021. Neuromuscul. Disord..

[26] Gasparotto M., Gatto M., Saccon F., Ghirardello A., Iaccarino L., Doria A. (2019). Pulmonary involvement in antisynthetase syndrome. Curr. Opin. Rheumatol..

[27] Ghirardello A., Gatto M., Franco C., Zanatta E., Padoan R., Ienna L., Gallo N., Zen M., Lundberg I.E., Mahler M. (2023). Detection of Myositis Autoantibodies by Multi-Analytic Immunoassays in a Large Multicenter Cohort of Patients with Definite Idiopathic Inflammatory Myopathies. Diagnostics.

[28] Moll S.A., Platenburg M., Platteel A.C.M., Vorselaars A.D.M., Janssen Bonas M., Kraaijvanger R., Roodenburg-Benschop C., Meek B., van Moorsel C.H.M., Grutters J.C. (2022). Prevalence and clinical associations of myositis antibodies in a large cohort of interstitial lung diseases. PLoS ONE.

[29] Satoh M., Tanaka S., Ceribelli A., Calise S.J., Chan E.K. (2017). A Comprehensive Overview on Myositis-Specific Antibodies: New and Old Biomarkers in Idiopathic Inflammatory Myopathy. Clin. Rev. Allergy Immunol..

[30] Foris V., Kovacs G., Matucci-Cerinic M., Olschewski H. (2013). PL-7 positive antisynthetase syndrome and pulmonary hypertension. J. Rheumatol..

[31] Hervier B., Meyer A., Dieval C., Uzunhan Y., Devilliers H., Launay D., Canuet M., Tetu L., Agard C., Sibilia J. (2013). Pulmonary hypertension in antisynthetase syndrome: Prevalence, aetiology and survival. Eur. Respir. J..

[32] Brown A.N., Strange C., Baughman R.P., Carbone R.G., Bottino G. (2009). Connective Tissue Disease and Vasculitis-Associated Interstitial Lung Disease. Pulmonary Arterial Hypertension and Interstitial Lung Diseases: A Clinical Guide.

[33] Sanges S., Yelnik C.M., Sitbon O., Benveniste O., Mariampillai K., Phillips-Houlbracq M., Pison C., Deligny C., Inamo J., Cottin V. (2016). Pulmonary arterial hypertension in idiopathic inflammatory myopathies: Data from the French pulmonary hypertension registry and review of the literature. Medicine.

[34] Lega J.C., Reynaud Q., Belot A., Fabien N., Durieu I., Cottin V. (2015). Idiopathic inflammatory myopathies and the lung. Eur. Respir. Rev..

[35] Ghigna M.R., Mooi W.J., Grunberg K. (2017). Pulmonary hypertensive vasculopathy in parenchymal lung diseases and/or hypoxia: Number 1 in the Series “Pathology for the clinician” Edited by Peter Dorfmuller and Alberto Cavazza. Eur. Respir. Rev..

[36] Dhont S., Zwaenepoel B., Vandecasteele E., Brusselle G., De Pauw M. (2022). Pulmonary hypertension in interstitial lung disease: An area of unmet clinical need. ERJ Open Res..

[37] Platteel A.C.M., Wevers B.A., Lim J., Bakker J.A., Bontkes H.J., Curvers J., Damoiseaux J., Heron M., de Kort G., Limper M. (2019). Frequencies and clinical associations of myositis-related antibodies in The Netherlands: A one-year survey of all Dutch patients. J. Transl. Autoimmun..

[38] Vulsteke J.B., De Langhe E., Claeys K.G., Dillaerts D., Poesen K., Lenaerts J., Westhovens R., Van Damme P., Blockmans D., De Haes P. (2019). Detection of myositis-specific antibodies. Ann. Rheum. Dis..

[39] Espinosa-Ortega F., Holmqvist M., Alexanderson H., Storfors H., Mimori T., Lundberg I.E., Ronnelid J. (2019). Comparison of autoantibody specificities tested by a line blot assay and immunoprecipitation-based algorithm in patients with idiopathic inflammatory myopathies. Ann. Rheum. Dis..

[40] Garcia-Fernandez A., Quezada-Loaiza C.A., de la Puente-Bujidos C. (2021). Antisynthetase syndrome and pulmonary hypertension: Report of two cases and review of the literature. Mod. Rheumatol. Case Rep..

[41] Lopes A.J., Capone D., Mogami R., Lanzillotti R.S., Melo P.L., Jansen J.M. (2011). Severity classification for idiopathic pulmonary fibrosis by using fuzzy logic. Clinics.

[42] Parikh R., Konstantinidis I., O’Sullivan D.M., Farber H.W. (2022). Pulmonary hypertension in patients with interstitial lung disease: A tool for early detection. Pulm. Circ..

[43] Parikh R., O’Sullivan D.M., Farber H.W. (2023). The PH-ILD Detection tool: External validation and use in patients with ILD. Pulm. Circ..

